# Recent outbreak of cutaneous anthrax in Bangladesh: clinico-demographic profile and treatment outcome of cases attended at Rajshahi Medical College Hospital

**DOI:** 10.1186/1756-0500-5-464

**Published:** 2012-08-28

**Authors:** Muhammad Afsar Siddiqui, Md Azraf Hossain Khan, Sk Shamim Ahmed, Kazi Selim Anwar, Shaikh Md Akhtaruzzaman, Md Abdus Salam

**Affiliations:** 1 Department of Dermatology, Rajshahi Medical College, Rajshahi 6000, Bangladesh; 2 Department of Microbiology, Rajshahi Medical College, Rajshahi 6000, Bangladesh; 3 Centre for injury Prevention and Research Bangladesh (CIPRB), Dhaka, Bangladesh

**Keywords:** Cutaneous anthrax, Clinico-demographic profile, Therapeutic response, Bangladesh

## Abstract

**Background:**

Human cutaneous anthrax results from skin exposure to *B. anthracis*, primarily due to occupational exposure. Bangladesh has experienced a number of outbreaks of cutaneous anthrax in recent years. The last episode occurred from April to August, 2011 and created mass havoc due to its dreadful clinical outcome and socio-cultural consequences. We report here the clinico-demographic profile and treatment outcome of 15 cutaneous anthrax cases attended at the Dermatology Outpatient Department of Rajshahi Medical College Hospital, Bangladesh between April and August, 2011 with an aim to create awareness for early case detection and management.

**Findings:**

Anthrax was suspected primarily based on cutaneous manifestations of typical non-tender ulcer with black eschar, with or without oedema, and a history of butchering, or dressing/washing of cattle/goat or their meat. Diagnosis was established by demonstration of large gram-positive rods, typically resembling *B. anthracis* under light microscope where possible and also by ascertaining therapeutic success. The mean age of cases was 21.4 years (ranging from 3 to 46 years), 7 (46.7%) being males and 8 (53.3%) females. The majority of cases were from lower middle socioeconomic status. Types of exposures included butchering (20%), contact with raw meat (46.7%), and live animals (33.3%). Malignant pustule was present in upper extremity, both extremities, face, and trunk at frequencies of 11 (73.3%), 2 (13.3%), 1 (6.7%) and 1 (6.7%) respectively. Eight (53.3%) patients presented with fever, 7 (46.7%) had localized oedema and 5 (33.3%) had regional lymphadenopathy. Anthrax was confirmed in 13 (86.7%) cases by demonstration of gram-positive rods. All cases were cured with 2 months oral ciprofloxacin combined with flucoxacillin for 2 weeks.

**Conclusions:**

We present the findings from this series of cases to reinforce the criteria for clinical diagnosis and to urge prompt therapeutic measures to treat cutaneous anthrax successfully to eliminate the unnecessary panic of anthrax.

## Background

Anthrax is a zoonotic disease of antiquity caused by *Bacillus anthracis*, an aerobic, spore-forming, large gram-positive rod [1]. The incidence of anthrax infection is diminishing in developed countries; however, it still remains a public health problem in developing countries, especially in areas where farming is the main source of income. Soil is contaminated with anthrax spores from the carcasses of dead animals and spores can survive for decades, even under adverse conditions, to serve as a source of infection for animals [2]. Humans are relatively resistant to cutaneous invasion, but the organisms may gain access through microscopic or gross breaks in the skin by contact with infected animals or their products like meat, hides, hair and bristles. There are three main forms of human anthrax, depending on the route of exposure: cutaneous, gastrointestinal and pulmonary or inhalational [3]. Cutaneous forms account for 95% of anthrax worldwide [4] and is characterized by rapidly developing necrotizing painless eschar (malignant pustule) with suppurative regional adenitis. Cutaneous infection starts as one or more painless, itchy papules or vesicles on the skin, typically on exposed areas such as the face, neck, forearms or hands. Within 7-10 days of the initial lesion, the papule forms an ulcer. The ulcer subsequently crusts over, forming a painless black eschar that is the hallmark of cutaneous anthrax. In addition, localized swelling, painful swollen regional lymph nodes and systemic symptoms can occur [5]. There is no report of direct human-to-human transmission in the literature and also there is no racial, sexual, or age predilection for anthrax. However, because anthrax is often related to industrial exposures and farming, the disease most often affects young and middle-aged adults. Death is rare with appropriate therapy, but untreated, the case fatality rate may reach up to 20%.

Anthrax was described in the early literature of the Greeks, Romans, Egyptians, and Hindus. The term *anthrakis* means coal in Greek, and the disease is named after the black appearance of its cutaneous form [6]. Until the twentieth century, anthrax infections killed hundreds of thousands of animals and people each year in Australia, Asia, Africa, North America, and Europe, particularly in the concentration camps during World War II [7]. Today there is concern about the use of anthrax as a biologic warfare agent. The disease is more common in developing countries without widespread veterinary or human public health programs.

Anthrax was reported in Bangladesh from 1980 to 1984 affecting both cattle and man [8], but it re-emerged in 2009-2010 with wider involvement. The animal anthrax, locally known as *‘Torka’,* is believed to have been enzootic in Bangladesh for a long time, and historically human outbreaks were always preceded by animal outbreaks. The Government of Bangladesh declared a red alert due to a sudden explosive outbreak of anthrax in 2010 that hit 12 districts and affected 607 people. The outbreak was investigated and thought to have been caused by the slaughter of infected cattle and selling or eating contaminated meat. The outbreak was most prevalent in the districts of Pabna, Sirajganj, Rajshahi, Kushtia and Tangail, which have greater cattle populations [9-11].

Health and livestock officials in Bangladesh have expressed great concern over a fresh outbreak of human anthrax prevailed from April to September 2011, mostly affecting two North-Western districts of Sirajganj (61 cases) and Pabna (32 cases). Additionally, districts of Bogra, Meherpur and Tangail had 28, 39 and 14 cases of anthrax respectively. Due to vaccine coverage in the preceding years, the number of livestock deaths was minimal and only a few infected slaughtered animals were held responsible for human transmission during the 2011 outbreak [12]. Fortunately there were no anthrax-related human deaths but meat sales drastically declined due to a lack of consumer confidence, and anthrax created mass havoc with significant economic losses related to cattle farming. Although there was no known case fatality, people panicked and mass immunization of livestock was demanded by concerned sections. Considering the overall impact of the recent outbreak of human cutaneous anthrax in Bangladesh, this study was conducted to examine its clinico-demographic profile and treatment outcomes of the affected population with the aim to generate awareness regarding the disease.

## Methods

This was a descriptive cross-sectional study of cutaneous anthrax patients during a recent outbreak in Bangladesh. Fifteen patients of different ages and sex were identified as cutaneous anthrax cases between April and August 2011 at the Outpatient Department of Dermatology of Rajshahi Medical College Hospital. The hospital is a tertiary care teaching facility situated in the northern part of Bangladesh which serves the main avenue for hospital care for the majority of people in the north and north-western parts of the country. The study was ethically approved by the Ethical Review Committee of Rajshahi Medical College, and informed consent was obtained from patients or a legal guardian in case of minor for their voluntary participation and publication of images in case of dissemination of scientific knowledge. Relevant clinical and socio-demographic data were collected through personal interview and clinical examination and recorded systematically. Strong clinical suspicion of cutaneous anthrax arouse among attending doctors during clinical examinations of patients presenting with very typical characteristics of anthrax ulcer, as described above. A patient history of recent contact with animal(s), alive or dead or butchering, dressing or washing of animal meat was noted as very pertinent information to substantiate the clinical diagnosis. Gram-stained smears prepared from aseptically collected ulcer exudates were examined for all cases under oil-immersion light microscope (Olympus CH-20, Japan). Demonstration of large gram positive bacilli, occurring singly or in short chains often with squared-off ends (safety-pin appearance) typically resembling to *B. anthracis,* was taken as laboratory evidence of cutaneous anthrax. Laboratory staff handling specimens from clinically suspected persons took safety measures including wearing surgical gloves, protective gowns and shoe covers. Every effort was made to avoid splashing or creating an aerosol, and protective eye wear and masks were worn. All potentially contaminated equipment/materials were decontaminated immediately by covering liberally with 5% hypochlorite and soaking for 30 min, then wiping with absorbent material soaked in disinfectant. All biohazardous waste was decontaminated by autoclaving [13]. Treatment of all 15 cases started by prescribing a combination of antibiotics, flucloxacillin (50 mg/kg/day) for 2 weeks and ciprofloxacin (20-30 mg/kg/day) for 2 months [14]. For patients in the paediatric age group, a suspension form of antibiotics was used, keeping in mind concerns about the development of arthropathy due to ciprofloxacin [15]. All patients were closely monitored for therapeutic response within the treatment period.

## Findings

All patients were treated and followed up as outpatients in the absence of any systemic involvement. Socio-demographical profile of cutaneous anthrax patients is shown in Table1. The mean age in years of the patients was 21.4 with ages ranging from 3 to 46 years. Out of 15 patients, 7 (46.7%) were male and 8 (53.3%) were female with a male to female ratio of 1:1.14. The majority of patients (80%) were from lower middle socioeconomic status (monthly income in BD Tk. 5001-10000). The overwhelming majority (86.7%) had an education level of primary school or below. Patients’ occupations were distributed into 4 categories: housewife, cultivator, butcher and pre-school/school with frequencies of 33.3%, 33.3%, 20.0% and 13.4% respectively. Regarding modes of acquisition of anthrax, contact with raw meat was the highest (46.7%), followed by contact with live animal (33.3%) and butchering (20%).

**Table 1:** Socio-demographical profile of cutaneous anthrax patients (n = 15)

	**n (%)**
Mean age in years (min-max)	21.4 (3-46)
Gender	
*Male*	7 (46.7)
*Female*	8 (53.3)
*M:F ratio*	1:1.4
Socioeconomic class	
*Lower class*	3 (20)
*Lower middle class*	12 (80)
Education	
*Pre school*	2 (13.3)
*Primary*	11 (73.4)
*Secondary*	2 (13.3)
Occupation	
*Pre school and School*	2 (13.4)
*Housewife*	5 (33.3)
*Butcher*	3 (20)
*Cultivator*	5 (33.3)
Modes of contact	
*Butchering*	3(20)
*Contact with raw meat*	7 (46.7)
*Contact with live animal*	5 (33.3)

**Figure 1:**
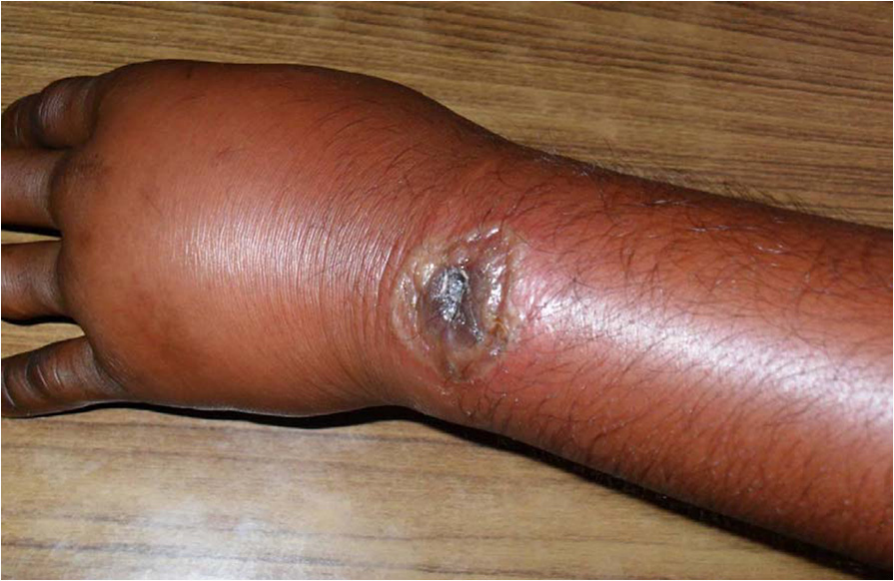
Cutaneous anthrax ulcer on the wrist with marked oedema.

**Figure 2:**
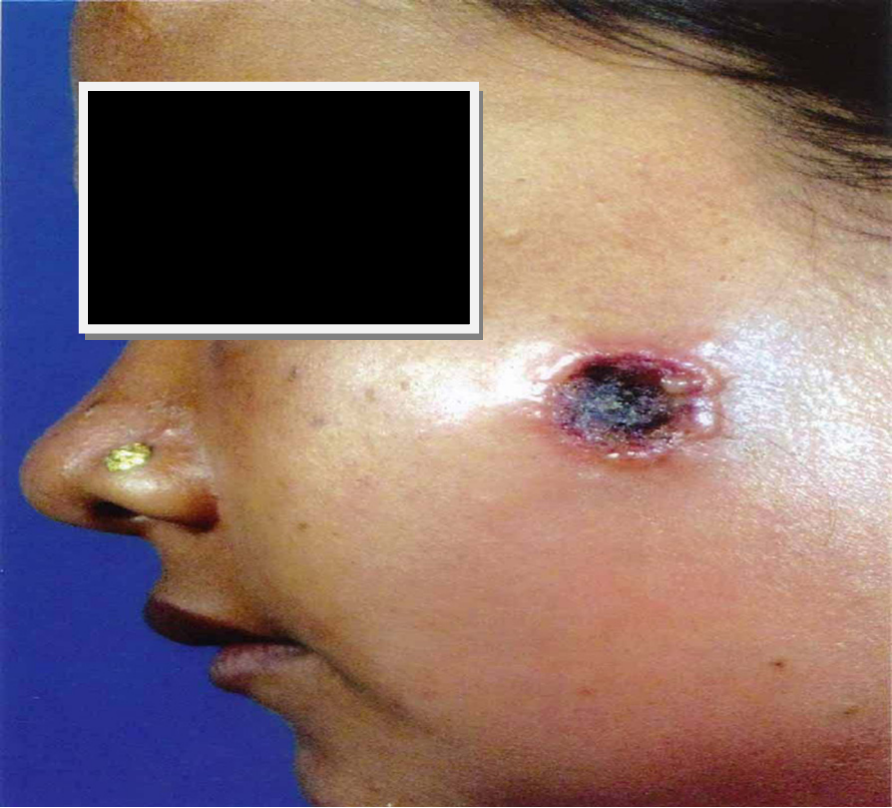
Cutaneous anthrax with black eschar and oedema on the face.

**Figure 3:**
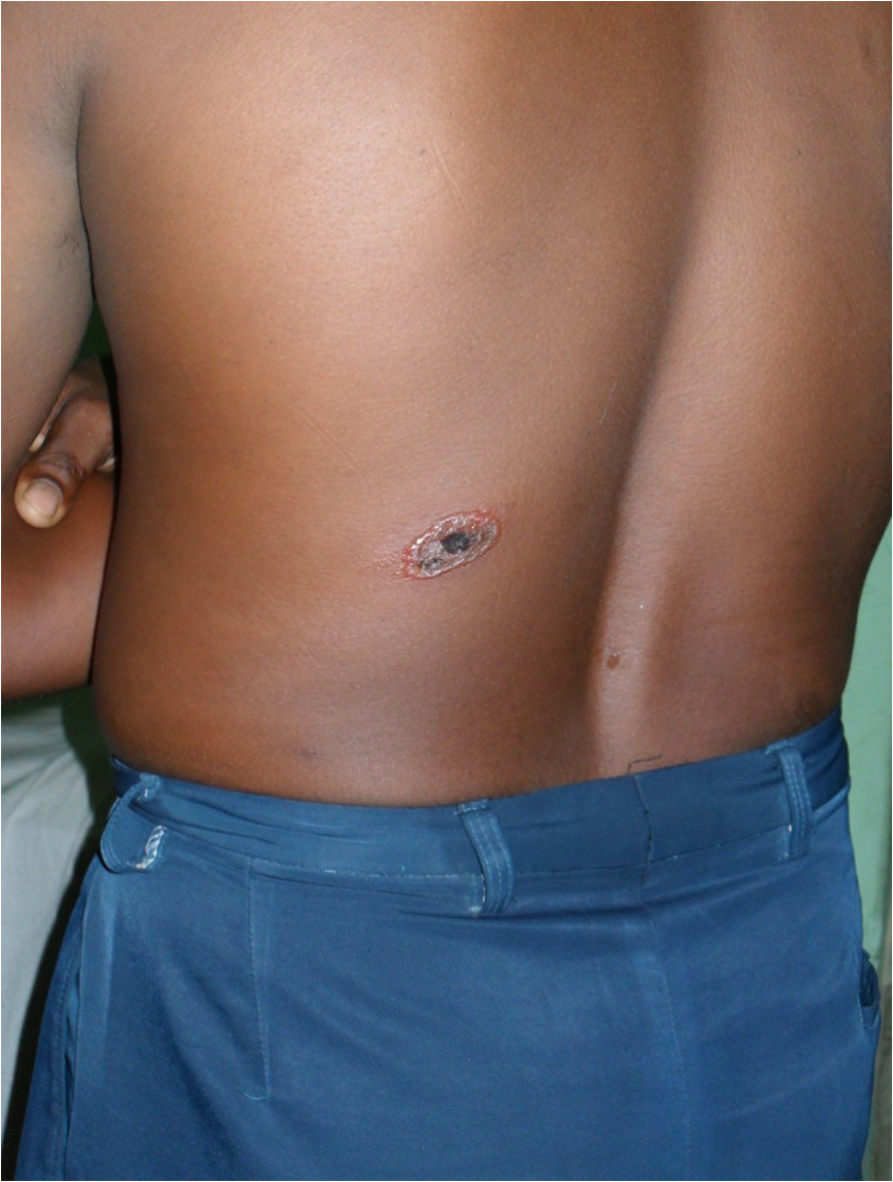
Cutaneous anthrax lesion with black eschar on back of the trunk.

Clinical presentations of cutaneous anthrax patients are summarized in Table 2. Patients presented with fever, localized oedema, regional lymphadenopathy and diarrhoea at a frequency of 8 (53.3%), 7 (46.7%), 5 (33.3%) and 2 (13.3%) respectively. In most of the cases (73.3%), ulcers were located on the upper extremity (Figure 1). Ulcer on the face (Figure 2) and trunk (Figure 3) was found in one case each. All patients were categorized into mild (80%) or moderate (20%) groups for their cutaneous presentations. Five (33.3%) patients gave a history of prior antibiotic therapy at diagnosis. Smear was found positive for gram-positive rods in 13 (86.7%) cases. All patients (100%) responded well and were cured with the treatment regimen.

**Table 2:** Clinical presentations, gram-staining findings and treatment outcome of anthrax patients (n=15)

	**n (%)**
Clinical features	
*Fever*	8 (53.3)
	
*Localized oedema*	7 (46.7)
*Regional lymphadenopathy*	5 (33.3)
*Diarrhoea*	2 (13.3)
Distribution of ulcer	
*Upper extremity*	11 (73.3)
*Both extremities*	2 (13.3)
*Face*	1 (6.7)
*Trunk*	1 (6.7)
Category of lesion	
*Mild (ulcer without oedema)*	12 (80)
*Moderate (ulcer with oedema)*	3 (20)
Prior antibiotic therapy	
*Antibiotic taken*	5 (33.3)
*Antibiotic not taken*	10 (66.7)
Gram-staining smear	
*Positive for gram-positive rods*	13 (86.7)
*Negative for gram-positive rods*	2 (13.3)
Treatment outcome	
*Cured*	15 (100)
*Not cured*	00 (00)

## Discussion

The ratio of human to animal anthrax cases in a country reflects the economic conditions, quality of surveillance, social traditions and dietary behaviour. Whereas in northern Europe, there has been one human infection per 10 livestock cases, in Africa and Asia there can be some 10 human cases per one livestock infection [3,16]. Although there is a routine anthrax vaccination programme for livestock in Bangladesh, unfortunately vaccination coverage is very low [17]. As a result, many animals acquire anthrax by ingestion of spores while grazing and the cycle of infection from carcass to grazing land continues. This cycle has resulted in anthrax being enzootic among livestock in Bangladesh. While reports of human anthrax in Bangladesh are relatively rare, because the disease is enzootic in agricultural settings and humans always get infection from infected animals, anthrax is likely to be prevalent in Bangladesh in endemic form.

Epidemiological investigations conducted for recent outbreaks in Bangladesh suggest that in each outbreak area, human cases started following slaughtering of anthrax-infected animals [18]. Accordingly, all anthrax cases in our series were also due to participation in slaughtering of anthrax-infected animals and handling, dressing or washing of infected meat. Females slightly outnumbered the males in our observation, which could be due to their exclusive involvement in dressing and washing of infected meat as a customary practice in Bangladesh. As far as the occupation, sites of distribution, prior antibiotic intake, and criteria for clinical diagnosis were concerned; our findings are very much in accordance with a recent study conducted in Turkey by Baykam et al. [19]. Microbiological diagnosis was confirmed for 13 of 15 cases (86.7%), which is consistent with study findings by Oncul et al. [20]. Two smear-negative cases (13.3%) can be explained by the inherent limitation of the procedure itself or history of prior antibiotic therapy. Although bacterial culture for isolation and polymerase chain reaction for nucleic acid detection are regarded as superior methods for laboratory diagnosis of anthrax, due to a lack of facilities, these methods could not be performed, which may also explain the discrepancy between clinical and laboratory diagnoses. All 15 patients were treated for anthrax despite two being smear-negative, but successful therapeutic response in all cases suggested that both smear-positive and negative cases were all anthrax patients. Although there was no sign of systemic involvement in any patient, all were treated with oral ciprofloxacin for 2 months as recommended for current management of cutaneous anthrax [14]. Use of ciprofloxacin in paediatric anthrax is not absolutely contraindicated but advocated with risk evaluation, and paediatric patients were closely monitored for any possible side effect. Oral flucloxacillin was also prescribed for 2 weeks for every patient based on benefit of doubt of any associated super infection caused by gram-positive coccus; a common pathogen causing skin and soft tissue infection.

It was revealed from careful history taking that, in most of the instances, the decision to slaughter a sick animal of suspected anthrax was influenced by economic considerations on the part of the animal owners, as well as the neighbors and other villagers who purchased the meat. Cattle owners slaughtered moribund animals to minimize financial loss as a result of the animal’s death, while local buyers, unaware of the risks associated with slaughtering, handling or eating meat from sick animals, purchased the relatively inexpensive infected meat. Demographic profile data show that poor education levels, poverty, and lacks of knowledge about the disease were all contributing factors for our patients who developed anthrax. Interestingly, clustering of cases was noted among members of the same family in a few instances, which correlated well with exposure to the same source of infection.

## Conclusions

A suspected human case of cutaneous anthrax can be diagnosed in any person who suffers from acute onset of skin lesions with papule or vesicle or skin ulceration with raised margin and central black eschar from the date of slaughtering the first sick animal in the outbreak area until three weeks after the last sick animal was slaughtered. Cutaneous anthrax is a curable condition with the use of proper antibiotics, so suspected and/or diagnosed cases must be brought under treatment to avoid unnecessary panic due to anthrax.

## Competing interests

The authors declare that they have no competing interests.

## Authors’ contributions

MAS and MAHK conceived the study, undertook clinical examinations and drafted the manuscript. SMA helped in data collection. KSA has done the critical review of the manuscript. SSA has contributed for laboratory diagnosis and MAS has made major contribution for intellectual thought, study design, statistical analysis and final revision and editing of the manuscript. All authors have read and approved the submitted version of manuscript.
